# “Heart in DRESS”: Cardiac Manifestations, Treatment and Outcome of Patients with Drug Reaction with Eosinophilia and Systemic Symptoms Syndrome: A Systematic Review

**DOI:** 10.3390/jcm11030704

**Published:** 2022-01-28

**Authors:** Milan Radovanovic, Djordje Jevtic, Andrew D. Calvin, Marija Petrovic, Margaret Paulson, Libardo Rueda Prada, Lawrence Sprecher, Ivana Savic, Igor Dumic

**Affiliations:** 1 Mayo Clinic Alix School of Medicine, Rochester, MN 55905, USA; Radovanovic.Milan@mayo.edu (M.R.); Calvin.Andrew@mayo.edu (A.D.C.); Paulson.Margaret@mayo.edu (M.P.); Prada.Libardo@mayo.edu (L.R.P.); Sprecher.Lawrence@mayo.edu (L.S.); 2 Department of Hospital Medicine, Mayo Clinic Health System, Eau Claire, WI 54703, USA; 3 School of Medicine, University of Belgrade, 11000 Belgrade, Serbia; djordje965@gmail.com (D.J.); ivanasavic86@yahoo.com (I.S.); 4 Department of Cardiovascular Medicine, Mayo Clinic Health System, Eau Claire, WI 54703, USA; 5 Icahn School of Medicine at Mount Sinai, New York, NY 10029, USA; petrovic_marija@yahoo.com

**Keywords:** myocarditis, heart failure, drug reaction, pericarditis, DRESS syndrome, drug hypersensitivity

## Abstract

Cardiac involvement in drug reaction with eosinophilia and systemic symptoms (DS) is rare but associated with high mortality. The aim of this research was to systematically review case reports by PRISMA guidelines in order to synthetize the knowledge of cardiac manifestations of DS. We identified 42 cases from 36 case reports. Women were two times more affected than men. Two-thirds of patients had cardiac manifestation in the initial phase of the disease, while in one-third of cases cardiac manifestations developed later (mean time of 70 ± 63 days). The most common inciting medications were minocycline (19%) and allopurinol (12%). In 17% of patients, the heart was the only internal organ affected, while the majority (83%) had at least one additional organ involved, most commonly the liver and the kidneys. Dyspnea (55%), cardiogenic shock (43%), chest pain (38%), and tachycardia (33%) were the most common cardiac signs and symptoms reported. Patients frequently had an abnormal ECG (71.4%), and a decrease in left ventricular ejection fraction was the most common echocardiographic finding (45%). Endomyocardial biopsy or histological examination at autopsy was performed in 52.4%, with the predominant finding being fulminant eosinophilic myocarditis with acute necrosis in 70% of those biopsied. All patients received immunosuppressive therapy with intravenous steroids, while non-responders were more likely to have received IVIG, cyclosporine, mycophenolate, and other steroid-sparing agents (60%). Gender and degree of left ventricular systolic dysfunction were not associated with outcomes, but short latency between drug exposure and the first DRESS symptom onset (<15 days) and older age (above 65 years) was associated with death. This underscores the potential importance of heightened awareness and early treatment.

## 1. Introduction

Drug reaction with eosinophilia and systemic symptoms (DRESS) syndrome (DS), formerly known as “drug-induced hypersensitivity syndrome” (DIHS) and “drug-induced delayed multiorgan hypersensitivity syndrome” (DIDMOHS) [1] is an idiosyncratic, and life-threatening drug reaction with complex pathophysiology that has not been completely elucidated. This fascinating syndrome has changed its nomenclature multiple times, reflecting our evolving understanding of the entity. Previous names including drug-induced pseudo-lymphoma and anticonvulsant hypersensitivity syndrome were derived from the syndrome’s typical characteristics: anticonvulsants are among the most common medications to cause the syndrome and lymphadenopathy is one of the most prominent features in addition to fever, rash, eosinophilia, and various visceral organ involvement [2,3,4]. Diagnosis of the syndrome requires a high index of suspicion with recognizing the temporal association between the plethora of its manifestations and exposure to the culprit medication. Exclusion of DRESS mimickers, mainly various infections, autoimmune disease, and neoplasm is mandatory.

Unlike other severe drug reactions which include cutaneous findings, the signature feature of DS is the involvement of internal organs, leading to the high mortality associated with this syndrome [2,3,4] and is felt to justify more aggressive treatment. While the liver is the most common visceral organ affected in DS, the heart, lungs, kidneys, intestines, pancreas, thyroid, and brain involvement have been described [2,3,4,5]. Mortality in DS depends on the extent of visceral organ involvement and death usually results from liver failure, respiratory failure, and/or fulminant myocarditis [6]. Mortality seems to be particularly high in patients who develop cardiac manifestations, with 50% of patients dying within 60 days from symptom onset [7].

The aim of this systematic review is to provide an update on cardiovascular manifestations of DS. To the best of our knowledge, this is the first systematic review of literature specifically analyzing all cardiac manifestations in patients with DS.

## 2. Materials and Methods

### 2.1. Definitions and Selection Criteria

We performed a systematic review of literature according to Preferred Reporting Items for Systematic Reviews and Meta-Analyzes (PRISMA) guidelines using the PubMed/Medline database from database inception until February 2021. We analyzed both DS cases and cases of eosinophilic myocarditis (EM) or giant-cell myocarditis (GCM) if they fulfilled the European Registry of Severe Cutaneous Adverse Reactions (RegiSCAR) scoring system criteria that have been used to define severe febrile drug eruptions.

Based on the RegiSCAR score, DS can be defined as no case (score < 2), possible case (score 2–3), probable case (score 4–5), and definitive case (score > 5) [4]. We selected only probable and definite DRESS cases based on the RegiSCAR score of 4 and above. In all cases, authors excluded alternative diagnoses, such as infectious, autoimmune diseases, and eosinophilic neoplasms.

Cardiac involvement was defined by symptoms of chest pain, shortness of breath (SOB), palpitations and/or abnormalities on electrocardiogram (ECG), cardiac biomarkers (i.e., troponin elevation), echocardiography findings and cardiac magnetic resonance imaging (cMRI) [8]. Cases of patients with histologically proven EM or GCM that fulfilled at least “probable” RegiSCAR criteria (i.e., a score of at least 4) were also included and analyzed.

Latency period was defined as the period (in days) from taking the culprit medication until the first DRESS symptom(s) development. Latency to cardiac symptoms was defined as the time from DS diagnosis to the occurrence of cardiac manifestations (if they were not part of the initial clinical presentation). Cut off to defining older age was 65 years.

### 2.2. Database and the Key Words (MeSH)

The following keywords alone and/or in combination were used searching PubMed/Medline database: “DRESS and heart”, “DRESS and cardiac”, “DRESS and coronary”, “DRESS and endocarditis”, “DRESS and myocarditis”, “DRESS and pericarditis”, “DRESS and pericardium”, “DIHS and heart”, “DIHS and cardiac”, “DIHS and coronary”, “DIHS and endocarditis”, “DIHS and myocarditis”, “DIHS and pericarditis”, and finally “DIHS and pericardium”. This search yielded 581 articles. “DIDMOHS” alone was searched, and it only yielded two articles, out of which none met the criteria for inclusion in our review.

A separate search was conducted with the keywords “eosinophilic myocarditis” alone and in combination with “drug hypersensitivity myocarditis” or “medication hypersensitivity myocarditis”, as well as “giant cell myocarditis” and “hypersensitivity”. These searches yielded an additional 818 and 21 articles, respectively. All these articles were further analyzed by the same authors using the same criteria. The cases that could represent DS with a score of at least 4 on the RegiSCAR scoring system, were included in the final review. We excluded cases where patients had a preexisting advanced heart failure and case reports/series of post-cardiac transplant, as these patients have been noted to have an increased incidence of EM, particularly in the setting of prolonged treatment with dobutamine and/or milrinone infusions, which are known causes of eosinophilia [9].

Duplicate articles, articles in languages other than English, pediatric cases, narrative reviews, abstracts, cases of DS without cardiac involvement, EM, and GCM cases that did not fulfil RegiSCAR criteria for probable/definite case, were all excluded. Finally, our review included 42 cases that fulfilled the above-set criteria.

**Figure 1:**
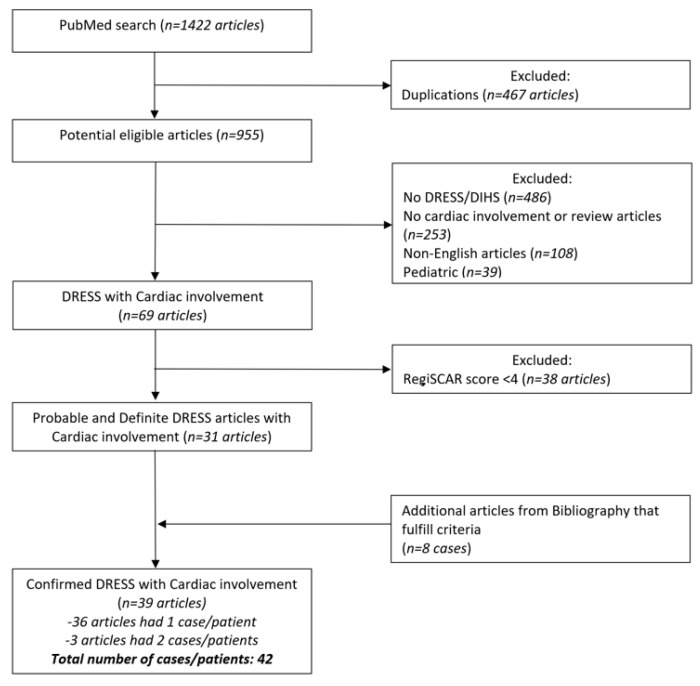
Figure one illustrates detailed flow chart of literature search according to PRISMA (Preferred Reporting Items for Systematic Reviews and Meta-Analyzes) guidelines.

The flow chart of the selection of the final cases included in the analysis is illustrated in Figure 1.

### 2.3. Data Collection

Two authors (M.R. and D.J.) independently and blindly identified and selected titles, abstracts, and full texts in the database search. Discrepancies of the selected articles were resolved by the senior author (I.D.). Subsequently, the reference list of selected articles was searched to identify any additional articles for inclusion, in accordance with previously established selection criteria. An Excel table was constructed, and for each case, we extracted patients’ demographic data, co-morbid conditions, immunosuppression, type of cardiac involvement, ECG findings, echocardiogram, cMRI, endomyocardial (EMB) and skin biopsy findings (if performed), the severity of eosinophilia, viral reactivation (Human Herpesvirus 6 (HHV-6) and Epstein-Barr virus (EBV)), other visceral organ involvement, culprit medication(s), latency period, treatment administered, and outcomes.

### 2.4. Statistical Analysis

Data were analyzed by descriptive and analytical statistics using SPSS statistical software (version 21.0) and expressed as mean ± standard deviation for continuous data, or as frequency and percentages for categorical data. The student’s *t*-test and chi-square test were used to compare data between two specific interest groups. Univariate regression analysis was used to determine factors associated with mortality in patients with DS. Statistical significance was reported using *p* value < 0.05.

## 3. Results

### 3.1. Demographics and Co-Morbidities

Our systematic review identified 42 unique patients from 36 case reports describing a single patient and 3 case series that described 2 patients each [10,11,12,13,14,15,16,17,18,19,20,21,22,23,24,25,26,27,28,29,30,31,32,33,34,35,36,37,38,39,40,41,42,43,44,45,46,47,48]. The age of patients in this systematic review ranged from 19–78 years for women (mean 41.8 years) and 22–84 years (mean 44.5 years) for men. Female patients were almost two times (61.9%) more affected compared to men (38.1%, Table 1), however, mortality was not associated with gender (Table 2). Patients’ aged above 65 was associated with worse outcomes.

The most common co-morbidities were rheumatologic (combined 21.4%) and hypertension (16.7%). Only one patient was immunosuppressed because of therapy for chronic prednisone for rheumatoid arthritis [30]. The presence of co-morbidities and the use of immunosuppression were not associated with patients’ outcomes (Table 2).

### 3.2. Presentation and Latency Period

The most common symptoms in cardiac DRESS were dyspnea (54.8%), hypotension or shock (42.9%), followed by chest pain (38.1%) and tachycardia (33.4%). Other arrhythmias (21.4%), cardiac arrest (11.9%), and pericardial effusion (9.5%) were less common.

About two-thirds of patients had recognized cardiac involvement at the time of presentation (69%), while one-third (29%) developed cardiac involvement only later in the course of the disease, with a latent period ranging from 13 to 371 days (mean 70.1 ± 63, median 55 days). One case didn’t have a reported timing of cardiac symptoms onset (38). Multi-organ involvement was common (35 of 42, 83.3%), with the most frequently affected visceral organs being the liver (32 of 42, 76.2%) and kidneys (10 of 42, 23.8%); cardiac-only involvement occurred in only 7 out of 42 patients (16.7%) (Table 1). However, the involvement of other visceral organs (in addition to the heart) didn’t clearly influence prognosis (*p* > 0.01).

The mean latency (defined as time-to-onset of DS symptoms) for patients who survived was 40.7 ± 23 days, while for patients who died was 26.7 ± 18 days (*p* < 0.05). Patients who had a shorter latency period (within 2 weeks, *p* = 0.013) had worse outcomes (Table 1 and Table 2).

### 3.3. Putative Causative Agents

The most commonly cited culprit medication was minocycline (19%) followed by allopurinol (12%), dapsone and sulfasalazine (7.1%) and lamotrigine (4.8%, Table 3). There were nine cases that had two or three suspected culprit medications that couldn’t be differentiated, and one case had four medications in differential [30].

### 3.4. Cardiac Evaluation

There were reported ECG abnormalities in the majority of patients (30 out of 42, 71.4%), with the most common abnormalities being sinus tachycardia (14 of 42, 33.4%), ST-segment elevation with and without T wave inversions (11 and 5 of 42, 26.2% and 11.9%, respectively), and bundle branch blocks (8 of 42, 19%). There were three reported cases of ventricular arrhythmias and two cases of high-grade atrio-ventricular heart block requiring permanent pacing. The most common reported echocardiographic finding was a reduced LVEF of less than 50% (19 of 42, 45.2%) followed by pericardial effusion (17 of 42, 40.5%). Regional wall motion abnormalities were seen in 11 out of 42 patients (26.2%). Slightly more than half of our analyzed patients had elevated cardiac biomarkers (24 of 42, 57%), from creatine kinase-MB (CK-MB) in case reports from the 1980s and 1990s to high-sensitive troponin in most recent articles. Only three cases had documented normal troponin despite cardiac involvement [26,34,37], and about one-third of the cases (15 of 42, 35.7%) didn’t have cardiac biomarkers reported despite other evidence of cardiac involvement.

Cardiac MRI was obtained in only five patients (11.9%), two of them showing no changes [22,37], but three cases showed either delayed enhancement pattern or hyperenhancement, mainly of the sub-pericardium and mid-myocardium, which are suggestive of acute myocarditis [11,15,25]. We found that in 22 cases (52.4%) that had EMB or an autopsy done, histopathologic analysis showed significant EM (including Acute necrotizing EM (ANEM)), predominance over GCM (67.4% vs. 18.2%), and in only 2 cases (9.1%) there were microscopic findings consistent with a combination of EM and GCM [20,35].

### 3.5. Treatment and Outcome

All patients received systemic steroid therapy. Other immunosuppressive medications were administered to patients who had not responded to steroids or showed further clinical deterioration. Cyclosporine, mycophenolate, and intravenous immunoglobulins were the most common used non-steroid medications. Cardiac-specific therapies included mechanical circulatory support (MCS), with the most utilized intra-aortic balloon pump, followed by extracorporeal membrane oxygenation (ECMO) and left ventricular assist device (LVAD). Only one patient had a cardiac transplant, but the patient unfortunately expired due to primary graft failure [21].

All therapeutic options administered to the patients described in this review are in Table 4.

Overall mortality in our group of patients was 45% (19 out of 42).

During individual analysis of parameters of interest, univariate regression analysis resulted in the selection of age and latency as possible contributors to patients’ mortality (Nagelkerke R^2^ = 0.669, *p* = 0.003, X^2^ = 16.210) (Table 2).

## 4. Discussion

Cardiac manifestations of DS are felt to be rare, but also under-recognized and unfortunately often discovered post-mortem. Prior case reports and case series on cardiac involvement in DS are notably small, and our understanding of this entity is limited and based on case reports, case series and single-center retrospective studies, lacking clear evidence-based guidelines on management, and treatment options. Here we present the largest systematic review and summary of the literature.

Prior studies of DS have reported a relatively low incidence of cardiac involvement in DRESS (11 of 83 patients, 13.3%): Intarasupht et al. [7] described cardiac manifestations in 8 out of 41 patients (20%) diagnosed with DS in Bangkok, Thailand hospital over a 5-year period; Eshki et al. [36] conducted a retrospective study in France over the span of 12 years and found cardiac involvement in only 2 out 15 patients (13%) diagnosed with DS; Ang et al. [49] reported only 1 case of myocarditis out of 27 analyzed patients (4%) diagnosed with DIHS over a 5-year period in Singapore hospital.

While there is informative literature about EM [50,51,52] and GCM [21,53,54,55], there is limited data on myocarditis due to DS [56]. In fact, we have not found a comprehensive review of cardiac manifestations or histopathologic findings specifically in patients with diagnosed DS. In 2017, Brambatti et al. [50] published a comprehensive systematic review of 179 histologically proven EM cases, finding that EM was associated with hypersensitivity in 61 patients, out of which only 10 cases fulfilled criteria for DS [19,22,24,26,33,35,36,38,41,46].

### 4.1. Age and Sex

It does not appear that DS is associated with the extremes of age, which is not surprising given the idiosyncratic nature of this reaction. A recent systematic review on pulmonary manifestations of DS [6] did not find a clear association between DS and age, contrary to findings of the current study, which demonstrated that patients above 65 years have worse outcomes.

The general consensus is that DS has no sex predilections. Some studies reported female predominance [57,58], while others reported DS being more common in the male population [7]. Our data showed female predominance (61.9%), but mortality was not associated with gender.

### 4.2. Clinical Manifestations of Cardiac Involvement in DS—Rare Manifestation of a Rare Entity

One of the unique characteristics of DRESS myocarditis is its delayed onset, which might occur long after well-recognized DRESS features (such as a rash, fever, and eosinophilia) have resolved. Cardiac damage during DS might be asymptomatic and cardiac involvement might be evident only by abnormal cardiac biomarkers, an ECG and/or echocardiography or might present with arrhythmia, acute heart failure, and cardiac arrest [7,10].

We found that the most common symptom that patients with DRESS syndrome and cardiac involvement reported was dyspnea in 55%, similarly to findings of Morikawa and co-authors [14], followed by cardiogenic shock and chest pain, present in 43% and 38% of the cases, respectively. Some unusual presentations have been described in literature, such as isolated right heart failure [59], cardiac arrest [10], and even high degree heart block [34]. Non-specific ECG abnormalities (sinus tachycardia, non-specific T wave and ST-segment abnormalities) were common and consistent with earlier reports [14]. DRESS myocarditis can predispose to different kinds of potentially life-threatening brady- and tachy-arrhythmias that may occur at any stage of the disease [60]. Based on the current ESC classification, arrhythmias in myocarditis can be classified according to the different evolutionary stages of myocarditis to the acute (hot) inflammatory stage and chronic (cold) post- inflammatory stage [61]. Similarly, in DRESS, myocarditis arrhythmias can occur early in the disease or later in the course as inflammation might persist. Acute inflammation because of adverse drug reactions might lead to pro-arrhythmogenic activity. Sudden cardiac death is more commonly associated with GCM [60]. It is unknown if patients with DRESS myocarditis continued to have pro-arrhythmogenic activity after resolution of DS and whether this electric instability proceeded to the “cold” stage.

Echocardiographic findings in these patients are various, ranging from mildly decreased LVEF to severe systolic dysfunction and/or pericardial effusion. In fact, 40% of patients with DS with heart involvement had pericardial effusion on echocardiogram and 7% underwent therapeutic pericardiocentesis [13,14,38]. Cardiac biomarkers are usually modestly elevated in hypersensitive myocarditis cases, as reported in Sabatine et al. [38].

As patients can lack cardiac symptoms during early evaluation, we suggest a baseline ECG and cardiac biomarkers be performed to actively screen for cardiac involvement. Those who are discovered to have abnormalities might benefit from cardiac telemetry and closer observation, given the high mortality associated with DS with cardiac involvement.

Whether the presence of co-morbid conditions contributes to the development of DS is unclear, and many questions remain unanswered regarding the influence of chronic cardiovascular conditions on the risk of its development. One previous review found that most of the patients who developed DS had hypertension, hyperlipidemia, and type 2 diabetes mellitus [7]. While the majority of the patients described in this review had no major chronic cardiac conditions, rheumatologic, and hypertension were the most common chronic condition.

In a univariate regression analysis, we did not find that patients who had chronic comorbidities were at a higher risk of dying (Table 2).

### 4.3. DRESS Myocarditis Histopathology

Histopathologically, myocarditis in DS manifests mostly as EM or GCM based on case reports in which biopsy was done [10,11,15,17,19,20,21,22,24,26,30,31,32,33,35,38,41,45,46,47,48]. EM is a rare form of myocardial inflammation characterized by an abundant eosinophilic infiltrate. It is associated with disorders such as DS, eosinophilic granulomatosis with polyangiitis (EGPA), idiopathic hypereosinophilic syndrome (HES), myeloproliferative disorders, parasitic infections, etc. [50]. EM clinical manifestations range from mild symptoms that can go unrecognized to fulminant cardiomyopathy and cardiogenic shock, rendering poor prognosis and high fatality rate. This fulminant form is known as ANEM [62]. We found that roughly half of our analyzed biopsy-proven ANEM cases had lethal outcomes (4 out of 9, 44%). Chronic forms are also well known and often complicated by restrictive cardiomyopathy (i.e., Loeffler endomyocarditis) [51]. While EM can occur in patients of various ages, GCM generally affects younger and previously healthy individuals, and it is rapidly progressive, with often a fatal clinical course [54,55]. GCM is characterized by diffuse or multifocal necrosis of cardiomyocytes, with inflammatory infiltrate consisting of lymphocytes, multinucleated giant cells, plasma cells, eosinophils, and rare neutrophils [53].

While EM is more common than GCM based on data from EMB from previous studies, it is important to note that diagnosis of GCM might be underrated due to its focal appearance and sampling error involved with conventional pathohistological evaluation [63,64]. Consequently, the number of proven GCM cases might be underestimated, and diagnosis can be significantly improved by using gene-expression profiling [63]. This technique refers to an analysis of specific genes responsible for the expression of time and disease-specific cytokines which are important inflammatory markers of GCM, and it can discover more than double the number of GCM compared to conventional histologic methods [63]. Hence, gene-expression profiling should be explored in future studies as a potentially useful method for diagnosing DRESS myocarditis.

### 4.4. Significance of Eosinophilia

Leukocytosis with eosinophilia and/or atypical lymphocytosis is almost universally present in DS. One recent study documented eosinophilia in more than 95% of cases [57]. It is unclear if the severity of eosinophilia has predictive value for outcome in DS. For example, a recent study [6] did not find an absolute eosinophil count (AEC) to be predictive of the severity of pulmonary manifestations of DS. Interestingly, the severity of eosinophilia was found to correlate with the end-organ damage in hypereosinophilic syndrome (HES) and some studies suggest that the severity of cardiac involvement depends on the duration and severity of eosinophilia [18,56]. In this review, AEC count was documented in the majority (80%) of cases and ranged from 270 to 9770 cells/mcL; however, the degree of eosinophilia was not associated with mortality.

### 4.5. Differential Diagnosis of Myocarditis Associated with Peripheral Eosinophilia

Differential diagnosis in patients who present with evidence of myocarditis, fever, and peripheral eosinophilia is broad. It includes a broad category of hypersensitivity/allergic reactions, idiopathic conditions such as HES, vasculitis (i.e., Eosinophilic granulomatosis with polyangiitis—EGPA), malignancies, a vast array of parasitic infections, tropical endomyocardial fibrosis, and transplant rejections [18,52,56]. Most importantly, differential diagnosis considerations are listed in Table 5.

Differentiating between DRESS myocarditis and HES might prove to be particularly challenging. Unlike patients with DS, those with HES, in addition to a rash, might also develop angioedema, urticaria, and have chronically elevated eosinophil counts above 1500 cells/mcL [52]. While it is a prominent feature of DS, fever is less common in patients with HES. The heart is one of the most common organs affected by HES, in DRESS, however, it is rare [51,52,65].

Churg-Strauss syndrome (CSS) initially presents with allergic inflammation of the upper respiratory airways with asthma and/or nasal polyps and palpable purpura. Typically, cardiac involvement in CSS manifests as acute pericarditis with mild pericardial effusion, however, eosinophilic endomyocarditis, dilated cardiomyopathy (CM), and congestive heart failure have been described as well [66]. In cases of viral myocarditis, histopathological findings are useful. An abundance of lymphocytes and more prominent necrosis of myocytes is usually present [54]. Parasitic infections are important considerations, particularly in developing countries (Table 5). Parasitic myocarditis, such as caused by *Toxocaracanis*, may be suspected if granulomatous reaction with increased eosinophils is observed in the myocardium, and myocyte destruction develops due to migrating larvae and inflammation [67]. Tropical endomyocardial fibrosis is nearly identical in presentation to HES; apart from a geographical predilection for residents in tropical or subtropical regions, it has no age predilection, can affect children, and other visceral organs are not affected. Loeffler endocarditis is a rare condition related to hypereosinophilia. It is characterized by endomyocardial infiltration by degranulated eosinophils, consequent fibrosis, and thickening of the endocardium, along with a restriction of diastolic filling [68].

Hematologic malignancies, such as chronic eosinophilic leukemia and primitive T-lymphoid disorder, may mimic hypersensitivity drug-induced myocarditis. However, considering relatively limited and not highly specific features in an endomyocardial biopsy, clinicopathological correlation is required for definitive diagnosis. Bone marrow biopsy and FIP1L1–PDGFRA and BCR–ABL fusion molecular testing, when available, are recommended to complete hematological workup [69].

### 4.6. Extent of Other Visceral Organs Involvement

The degree of visceral organ involvement has been described to correlate with mortality of DS and cardiac involvement, in particular, portends poor prognosis [7]. In one prospective study of patients with DS, both 30- and 90-day mortality was higher in those who had cardiac involvement [7]. In particular, the mortality rate of patients with cardiac involvement has been reported as 50% [7] and 44% [14]. We found that only in 7 out of 42 patients (16.7%) the heart was affected as the sole organ, compared to 35 patients (83.3%) that had at least one more internal organ involvement (mainly liver, followed by kidneys). Interestingly, we do not find any association between multi-organ involvement and outcome (*p* > 0.05).

### 4.7. Medications and Latency

Previous reports documented minocycline and ampicillin to be the most common causative agent associated with cardiac involvement in DS [8,14]. Similarly, we found that minocycline was the culprit in 19% of cases (8 of 42) followed by allopurinol in 11.9% (5 of 42). We didn’t find minocycline to have a significantly different rate or time to death compared to other medications (*p* > 0.05). The other implicated medications are presented in Table 3. It is important to note that due to polypharmacy, it was not always possible to decipher which medication was the exact cause of the syndrome. As illustrated in this review, 23.8% of patients received two or more medications that could be the culprit, and the exact offending medication was not determined.

The mean time from medication administration to development of the first cardiac symptoms, in this review was found to be 70.1 ± 63 days (ranging from 13–371). As we can see, 28.6% of patients developed cardiac complications later in the course of DS after other, easier to recognize manifestations have already occurred. Hence, it is important to counsel and educate patients who are diagnosed with DS about potential cardiac complications that can occur later during the disease course. The onset of cardiac symptoms was not related to the patients’ mortality (*p* > 0.05). However, our review analysis shows a worse prognosis in patients with earlier DS symptoms onset. Latency, defined as time-to-onset of DS symptoms, was shorter in patients who died, especially if symptoms started within two weeks after culprit medication ingestion (*p* = 0.013). A recent paper described shorter latency in patients who were treated with amoxicillin, postulating that viral reactivation triggered by amoxicillin might be responsible [70].

### 4.8. Treatment and Outcome

Symptomatic treatment with topical antihistaminic and steroid agents is sufficient for DS affecting the skin and without visceral involvement. However, if there is cardiac involvement, systemic immunosuppressive therapy is necessary. While some authors hypothesized that systemic steroid administration might exacerbate the syndrome in the case of augmented viral replication, multiple reports of improvement with immunosuppressive regimens and the fact that steroid withdrawal is associated with relapse of the disease argue against this theory. Some authors suggested that delays in immunosuppressive medication administration are associated with worse outcomes [71]. Novel non-steroid medications, such as Tofacitinib or Rituximab, have been used successfully in patients who had recurrent relapses with steroid tapering [11].

In this systematic review, all patients received systemic steroid therapy. Other immunosuppressive medications were administered to patients who had not responded to steroids or showed further clinical deterioration. Cyclosporine, mycophenolate, and intravenous immunoglobulins were the most common steroid-sparing regimens prescribed (Table 4). Novel steroid-sparing options, such as IL-5 blockers (mepolizumab), has been used successfully in patients who had recurrent relapses with steroid tapering [15]. Due to the rarity of this syndrome and different clinical practices around the world, there is no standard recommendation about the dose of steroids to be used or the speed and fashion in which steroids should be tapered. It would seem prudent to offer a prolonged taper when the offending agent has a particularly long half-life (i.e dapsone, amiodarone). Recurrences are not uncommon in such cases and might be associated with worse clinical outcomes. Progression from hypersensitivity myocarditis to ANEM during the episodes of recurrence has been described [15,72]. Some case reports reported better outcomes with a combination of immunosuppressive medications [14], however, we did not find a statistically significant difference in survival between patients who received only steroids and those who additionally received another immunosuppressant.

Extracorporeal membrane oxygenation (ECMO) has been used successfully in patients with prolonged and refractory cardiogenic shock [10]. It is important to keep in mind that high doses of steroids have been associated with the development of arrhythmias in some cases, although it remains rare [34]. Standard pharmacotherapy for patients with reduced LVEF, including beta-blockers, angiotensin-converting enzyme inhibitors/angiotensin receptor blockers, neprolysin inhibitors, and aldosterone antagonists would seem reasonable and concordant with current guidelines [73].

Mortality in this selected group of patients with probable and definite DS who all had cardiac involvement was 45.2%, which is similarly high to the previous reports [14,56], but much higher than mortality of DS in general, around 10% [2].

### 4.9. Limitations of the Study

The limitation of our systematic review is that we have included only probable and definite cases of DS. Additionally, we have included only cases in English and ones that were published in journals that are indexed in the PubMed/MEDLINE database. While these strict criteria were implemented to avoid low-quality case reports, we recognize that we might have missed some high-quality cases if they did not meet our pre-selection criteria. As with every systematic review of case reports and case series, publication bias might influence our findings. Finally, the study sample is relatively small with 42 cases selected, which limits our findings. 

## 5. Conclusions

The most common symptoms in patients with DS and cardiac involvement are dyspnea and chest pain, while the most common culprit medications were minocycline and allopurinol. All patients had changes on ECG and/or echocardiography. Clinicians should consider performing EMB early to determine a diagnosis. Prognosis is highly variable, with no clear relationship to gender, co-morbidity, the severity of eosinophilia or degree of systolic dysfunction. The mortality of patients with cardiac involvement is extremely high (45.2%), and older age (above 65 years) and a short latency between medication exposure and symptom onset was associated with mortality. Patients with a cardiac manifestation of DS should be monitored on telemetry during inpatient management, particularly those older than 65 years and ones who exhibited shorter latency periods. The role of prolonged rhythm monitoring of these patients in outpatient settings remains unclear and should be further investigated. Further research, preferably multicenter prospective studies are also needed to determine optimal treatment and the role of adding mepolizumab to solumedrol as the first-line therapy given the poor outcome with current management strategies.

## Figures and Tables

**Table 1:** The epidemiology, demographics, clinical presentation, diagnostic findings, and outcome in DRESS syndrome cases.

Demographic Characteristics				
Gender			Age range (years)	Mean age (years)
	Female	26 (61.9%)	19–78	41.8
	Male	16 (38.1%)	22–84	44.5
Total		42 (100%)	19–84	42.9
Race				
	Not reported	25 (59.5%)		
	Asian	9 (21.4%)		
	Caucasian	5 (12%)		
	African-American	3 (7.1%)		
**Co-morbidities**				
None		19 (45.2%)		
Rheumatologic		9 (21.4%)		
Hypertension		7 (16.7%)		
Oncologic		3 (7.1%)		
Infectious		2 (4.8%)		
Other, less common (total)		8 (19%)		
**Visceral organs involved**				
Heart only		7 (16.7%)		
Heart and 1–2		30 (71.4%)		
Heart and 3 or more		5 (11.9%)		
**Clinical presentation**				
Arrhythmia		25 (59.5%)		
Dyspnea		23 (54.8%)		
Hypotension/Shock		18 (42.9%)		
Chest pain		16 (38.1%)		
Cardiac arrest		5 (11.9%)		
Syncope		3 (7.1%)		
**Timing of cardiac symptoms**				
On initial presentation		29 (69%)		
Delayed		12 (28.6%)		
Unknown		1 (2.4%)		
**Latency**		**Mean (days):**	**Range (days):**	**Statistical significance**
Drug to DRESS symptoms latency		34.7 ±22	5–91	***p* < 0.05**
	Recovered patients	40.7 ± 23	10–91	
	Patients who died	26.7 ± 18	5–61	
Drug to cardiac symptoms latency		70.1 ± 63	13–371	*p* > 0.05
	Recovered patients	58.7 ± 27	28–108	
	Patients who died	82.9 ± 22	13–371	
**ECG findings**				
Normal or not reported		12 (28.6%)		
Abnormal		30 (71.4%)		
	Sinus Tachycardia		14 (33.4%)	
	ST elevation		11 (26.2%)	
	Fascicular blocks (right and left)		8 (19%)	
	ST depression & T inversions		5 (11.9%)	
	Atrio-ventricular block		3 (7.1%)	
	Atrial fibrillation		3 (7.1%)	
	Ventricular arrhythmia		3 (7.1%)	
	Bradycardia		2 (4.8%)	
**Echocardiography findings**				
LVEF < 50% (mean: 27.05 ± 13.2%; range:10–50)		19 (45.2%)		
Pericardial effusion		17 (40.5%)		
Regional wall motion abnormalities		11 (26.2%)		
LV hypertrophy		4 (9.5%)		
**Cardiac MRI**				
Performed		5 (11.9%)		
	Delayed (hyper)enhancement		3 (60%)	
	Normal finding		2 (40%)	
**Histopathological examination**				
Endomyocardial biopsy/Autopsy		22 (52.4%)		
	ANEM		9 (40.1%)	
	Eosinophilic myocarditis		6 (27.3%)	
	GCM		4 (18.2%)	
	Mixed infiltrate		2 (9.1%)	
	Uncertain		1 (4.5%)	
**Outcome**				
Recovered		23 (54.8%)		
Death		19 (45.2%)		

LVEF—left ventricular ejection fraction; ANEM—acute necrotic eosinophilic myocarditis; GCM—giant-cell myocarditis.

**Table 2:** Regression analysis in prediction of patients’ mortality.

	Univariate Regression Analysis		
Variable	*p* Value	OR	95% CI for OR
Age	0.035	0.872	0.768–0.990
Sex	0.228	12.808	0.203–808.796
Comorbidities	0.266	0.022	0.000–18.361
Pulse	0.133	3.719	0.670–20.630
Allopurinol	0.119	6.581	0.614–70.524
Minocycline	0.196	0.296	0.047–1.874
Latency	**0.028**	1.162	1.016–1.139
AEC	0.125	1.001	1.000–1.002
LVEF	0.876	1.006	0.937–1.079

AEC—absolute eosinophil count; LVEF—left ventricular ejection fraction.

**Table 3:** List of the culprit medication in patients with DRESS syndrome who had cardiac manifestations.

Culprit Medication	Number of Cases
Minocycline	8 (19%)
Allopurinol	5 (11.9%)
Dapsone	3 (7.1%)
Sulfasalazine	3 (7.1%)
Lamotrigine	2 (4.8%)
Amoxicillin-Clavulanate, Azithromycin, Bisoprolol, Bupropion, Carbamazepine, Furosemide, Modafinil, Phenytoin, Chlorthalidone, Loxoprofen, Salazosulfapyridine	Each in 1 case (2.4%)
Suspected polypharmacy	10 (24%)
Ciprofloxacin vs. Scopolamine vs. Dipyrone vs. Diclofenac	
Anticonvulsants (Phenytoin, Valproic acid or Carbamazepine)	
Amitriptyline vs. Diclofenac vs. Lorazepam	
Captopril vs. Bisoprolol	
Cefaclor vs. NSAID	
Colchicine vs. Allopurinol	
Lithium vs. Quetiapine	
Phenobarbital vs. Phenytoin	
Phenobarbital vs. Phenytoin vs. Metharbital	
Trimethoprim/Sulfamethoxazole vs. Zonisamide	

NSAID—Nonsteroidal anti-inflammatory drug.

**Table 4:** Therapeutic options administered to the patients described in this review.

Therapeutic Options		Number of Cases
Immunomodulators		
Steroids		42 (100%)
Antihistamines		11 (26.2%)
Steroid sparing therapies		26 (61.9%)
	Cyclosporine	5 (11.9%)
	IVIG	5 (11.9%)
	Mycophenolate-mofetil	5 (11.9%)
	Methotrexate	2 (4.8%)
	Azathioprine	2 (4.8%)
	Tofacitinib	2 (4.8%)
	Colchicine	1 (2.4%)
	Mepolizumab	1 (2.4%)
	Rituximab	1 (2.4%)
	Plasmapheresis	1 (2.4%)
	OKT-3	1 (2.4%)
	Anti-thymocyte globulin	1 (2.4%)
Cardiac Specific therapies		
Vasopressors/Inotropic agents		9 (21.4%)
Intra-aortic balloon pump		5 (11.9%)
Pericardiocentesis		3 (7.1%)
ECMO		3 (7.1%)
LVAD		1 (2.4%)
Cardiac transplant		1 (2.4%)

IVIF—intravenous immunoglobulins; OKT-3—Muromonab-CD3; LVAD—left ventricular assisted device; ECMO- extracorporeal membrane oxygenation.

**Table 5:** The differential diagnosis for patients presenting with fever, eosinophilia, and evidence of cardiac involvement (either by symptoms and/or evidenced by an abnormality in electrocardiogram, cardiac enzymes, or echocardiography).

Disease Category	Specific Disease/Comment
Allergic/hypersensitivity reactions to medications	DRESS syndrome, smallpox vaccination, dobutamine
Infectious Disease	Parasitic infections (*Toxocara canis*, *Toxoplasma gondii*, *Trichinella spiralis*, *Trypanosoma cruzi*, *Echinococcus* spp., *Strongyloides stercoralis*)
Neoplastic	Leukemia and lymphoma
Paraneoplastic	Carcinoma of biliary tract and lungs
Idiopathic	Hypereosinophilic syndrome, Loeffler endomyocardial fibrosis, tropical endomyocardial fibrosis
Vasculitis	Eosinophilic granulomatosis with polyangiitis
Allograft rejection	Heart transplant

## Data Availability

All data are publicly available.
